# Persistence to anti-CGRP monoclonal antibodies and onabotulinumtoxinA among patients with migraine: a retrospective cohort study

**DOI:** 10.1186/s10194-023-01636-8

**Published:** 2023-08-02

**Authors:** Larry Charleston, Brian Talon, Christine Sullivan, Carlton Anderson, Steven Kymes, Stephane A. Regnier, Seema Soni-Brahmbhatt, Stephanie J. Nahas

**Affiliations:** 1 Michigan State University College of Human Medicine, MI East Lansing, USA; 2 Lundbeck LLC, IL Deerfield, USA; 3 H. Lundbeck A/S, Copenhagen, Denmark; 4 Department of Neurology, Thomas Jefferson University, Jefferson Headache Center, 900 Walnut Steet, Suite 200, PA 19107-5509 Philadelphia, USA

**Keywords:** Migraine, Persistence, onabotulinumtoxinA, Erenumab, Fremanezumab, Galcanezumab, Eptinezumab, Anti-CGRP monoclonal antibody, Real-world evidence

## Abstract

**Background:**

To date, real-world evidence on persistence to anti-calcitonin gene-related peptide (anti-CGRP) monoclonal antibodies (mAbs) or onabotulinumtoxinA have excluded eptinezumab. This retrospective cohort study was performed to compare treatment persistency among patients with migraine on anti-CGRP mAbs (erenumab, fremanezumab, galcanezumab, or eptinezumab) or onabotulinumtoxinA.

**Methods:**

This retrospective study used IQVIA PharmMetrics data. Adult patients with migraine treated with an anti-CGRP mAb or onabotulinumtoxinA who had 12 months of continuous insurance enrollment before starting treatment were included. A “most recent treatment episode” analysis was used in which the most recent episode was defined as the latest treatment period with the same drug (anti-CGRP mAb or onabotulinumtoxinA) without a ≥ 15-day gap in medication supply on/after June 25, 2020, to December 31, 2021. Patients were indexed at the start of their most recent episode. Patients were considered non-persistent and discontinued the therapy associated with their most recent episode if there was ≥ 15-day gap in medication supply. A Cox proportional-hazards model estimated the discontinuation hazard between treatments. The gap periods and cohort definition were varied in sensitivity analyses.

**Results:**

The study included 66,576 patients (median age 46 years, 88.6% female). More eptinezumab-treated patients had chronic migraine (727/1074), ≥ 3 previous acute (323/1074) or preventive (333/1074) therapies, and more prior treatment episodes (3) than other treatment groups. Based on a 15-day treatment gap, patients on subcutaneous anti-CGRP mAbs had a 32% (95% CI: 1.19, 1.49; erenumab), 42% (95% CI: 1.27, 1.61; galcanezumab), and 58% (95% CI: 1.42, 1.80; fremanezumab) higher discontinuation hazard than those receiving eptinezumab, with this relationship attenuated, but still statistically significant based on 30-day and 60-day treatment gaps. There was no significant difference in the discontinuation hazard between eptinezumab and onabotulinumtoxinA. Based on a 15-day treatment gap among patients who newly initiated therapy, the discontinuation hazard of subcutaneous anti-CGRP mAbs remained significantly higher compared to eptinezumab and onabotulinumtoxinA.

**Conclusion:**

Patients treated with eptinezumab demonstrated persistency that was higher than subcutaneous anti-CGRP mAbs and similar to onabotulinumtoxinA.

**Graphical Abstract:**

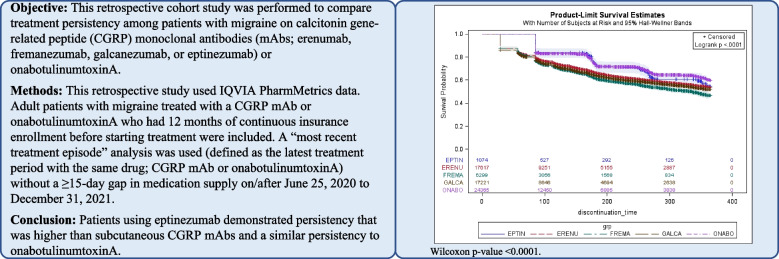

**Supplementary Information:**

The online version contains supplementary material available at 10.1186/s10194-023-01636-8.

## Introduction

The American Headache Society recommends preventive therapy primarily based on migraine attack frequency and degree of disability, but other factors (including failure of and over-reliance upon acute medications) should also be considered [1]. Traditional non–anti-calcitonin gene-related peptide (CGRP) oral preventive therapy for migraine includes antidepressant, anti-hypertensive, and anti-epileptic treatments. Persistence to such therapies is low—81% of patients have gaps of over 90 days in the first year of treatment, and approximately two-thirds of patients discontinue therapy after the first year [2]. Medication persistence is defined as the act of continuing a treatment for the entire duration prescribed [3]. Common reasons for discontinuation can include side effects and lack of efficacy [4].

Beyond traditional oral medications, advanced preventive therapies for migraine have emerged. These include onabotulinumtoxinA (delivered intramuscularly [IM]) [5] and a new class of preventive drugs, including eptinezumab (delivered intravenously [IV]) [6], fremanezumab [7], galcanezumab [8], and erenumab (all delivered subcutaneously [SC]) [9], all of which are monoclonal antibodies (mAbs) that either target CGRP or its receptor (in the case of erenumab). OnabotulinumtoxinA was approved by the U.S. Food and Drug Administration (FDA) in 2010 for the prevention of chronic migraine (CM) [10]. It is recommended for patients who have contraindications or intolerability to, or prior treatment failures from at least two traditional oral preventive medications. Anti-CGRP mAbs are often recommended if there is an inability to tolerate or a contraindication to oral non–anti-CGRP preventive therapies, after a failure of ≥ 2 oral therapies at an established potentially effective dose over a duration of 8 weeks, or after failure of two consecutive quarterly injections of onabotulinumtoxinA for CM [1].

To the best of our knowledge, comparative studies on persistence among all anti-CGRP mAb treatments and onabotulinumtoxinA have not been conducted. Therefore, the aim of this retrospective cohort study was to compare persistence among patients with migraine to SC-delivered erenumab, fremanezumab, and galcanezumab; IV-delivered eptinezumab; or IM onabotulinumtoxinA. As there is no established fixed treatment algorithm, this type of analysis only allows for assessing elements of comparative effectiveness.

## Methods

### Study design

This was an observational retrospective cohort study of United States claims data to measure the association between the type of preventive migraine treatment (i.e., SC erenumab, fremanezumab, and galcanezumab; IV eptinezumab; and IM onabotulinumtoxinA) and persistence to treatment. The overall study period was June 25, 2019, through December 31, 2021. The index period began on June 25, 2020. This date was selected because it was the date of the first eptinezumab claim and reflects when all five medications had claims available.

**Fig. 1:**
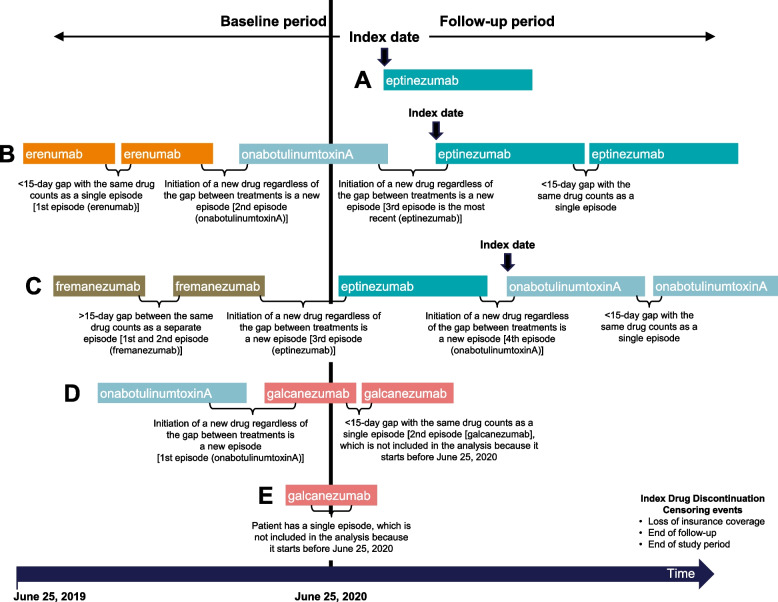
Study timeline and indexing. Scenario **A**: A patient has no history of anti-CGRP mAb nor onabotulinumtoxinA use and initiates a new preventive therapy (e.g., eptinezumab) after June 25, 2020. The patient is included in the eptinezumab treatment group and is indexed on the day of the eptinezumab claim. Scenario **B**: A patient begins with 2 periods of treatment (e.g., erenumab) which are < 15 days apart, which counts as a single episode (i.e., 1st episode). The patient then starts a new drug (e.g., onabotulinumtoxinA), which is a new treatment episode (2nd episode). The patient then initiates a new drug (e.g., eptinezumab) which is the start of the 3rd and most recent episode – the patient is indexed at the start of the new treatment (e.g., eptinezumab). Scenario **C**: A patient begins with 2 periods of treatment (e.g., fremanezumab) which are > 15 days apart, which count as distinct episodes (i.e., 1st and 2nd episodes). The patient subsequently initiates two new therapies (e.g., eptinezumab then onabotulinumtoxinA), in which onabotulinumtoxinA is the most recent episode. The patient is indexed at the start of the new therapy (e.g., onabotulinumtoxinA). Scenario **D** and **E**: The most recent episodes of treatment (e.g., galcanezumab) begin before June 25, 2020, and are therefore not included in the analysis

The base case analysis utilized a “most recent treatment episode analysis,” in which a treatment episode was defined as a period of treatment with the same drug during which the patient fills the prescription no more than 15 days after exhausting the days’ supply of the current treatment (Fig. 1). A treatment episode can be defined as a non-interrupted treatment sequence based on the treatment posology. Patients were indexed based on the first drug claim after June 25, 2020, which started their most recent treatment episode. Patients were followed from their index date until the first occurrence of the following events: discontinuation of the index therapy (primary outcome of interest), loss of medical or pharmacy insurance coverage (i.e., ≥ 1-month gap), end of the follow-up period (1 year), or end of the study period. Patients who may have lost medical or pharmacy insurance coverage (assumed based upon their eligibility file), reached the end of the follow-up period, or reached the end of the study period without discontinuing the index medication were right-censored (i.e., the discontinuation occurred after the study period and the exact discontinuation date is unknown).

### Data source and study population

The data source utilized was the IQVIA PharmMetrics Plus database, which contains claims data on more than 100 million commercially insured individuals with an age and geographic distribution similar to that of the United States. Claims occurring in both the inpatient and outpatient settings, as well as prescription claims from retail and mail-order pharmacies, were captured. Claims data included dates of services, associated diagnosis codes, and dollar amounts paid by insurers and patients. The study population consisted of patients with migraine receiving eptinezumab, erenumab, fremanezumab, galcanezumab, or onabotulinumtoxinA.

### Inclusion and exclusion criteria

The cohort consisted of patients with ≥ 1 prescription claim for erenumab, fremanezumab, galcanezumab, eptinezumab, or onabotulinumtoxinA in the most recent treatment episode occurring after June 25, 2020. Patients were divided into five treatment groups based on the index therapy (erenumab, fremanezumab, galcanezumab, eptinezumab, or onabotulinumtoxinA). Patients were ≥ 18 years of age on the index date, had ≥ 12 months of continuous medical and pharmacy coverage, and in order to establish a valid migraine diagnosis had ≥ 2 claims associated with a diagnosis code for migraine (International Classification of Diseases-10 [ICD-10] diagnosis code beginning with G43.XX) ≥ 30 days apart occurring in the inpatient or outpatient setting within 12 months prior to the index date. Patients with a diagnosis code for G43.7XX were classified as CM based on the associated diagnosis code on the medical claim closest to the index date. Patients who did not meet the above criteria, patients with missing covariate data, and patients with any diagnosis of cluster headache in the baseline period were also excluded.

### Study variables

The independent variable of interest was the type of preventive migraine therapy based on the drug used on the index date (erenumab, fremanezumab, galcanezumab, eptinezumab, or onabotulinumtoxinA). Covariates were defined a priori and included variables that could confound the association between treatment and discontinuation. Covariates were measured on the index date or within 12 months prior to the index date and included continuous (age, patient co-pay amount for preventive therapy), categorical (sex, region, payer type, migraine type [i.e., episodic vs chronic migraine], presence of select comorbidities, prescriber specialty, anti-CGRP mAb and onabotulinumtoxinA treatment history, history of oral preventive therapy use, history of acute therapy use, number of prior episodes), dichotomous (use of gepants), and discrete (Charlson comorbidity index, history of emergency department visits, history of urgent care visits, history of inpatient hospitalizations). Traditional oral preventive medications for migraine may be used for other chronic conditions. Therefore, we further required that the claim for such non anti-CGRP oral preventive medication be accompanied by a corresponding claim in the inpatient or outpatient setting associated with a migraine diagnosis code (ICD-10 G43.XX) on or within 14 days prior to the prescription claim, and that the medication have at least a 28-day supply [11]. Similarly, the use of an acute medication had to be accompanied by a corresponding claim in the inpatient or outpatient setting associated with a migraine diagnosis code on or within 7 days prior to the prescription claim.

The dependent variable was the time from the index date to the first occurrence of either index drug discontinuation or censoring. Discontinuation was defined in the base case as a 15-day gap in therapy beginning from the date after the last day of therapy as indicated by the days’ supply of the drug. If a patient received an early claim for an anti-CGRP SC mAb prior to exhaustion of the days’ supply of the prior claim, then the date of the early claim was shifted forward in time after completion of the prior claim’s days’ supply (assuming the patient will “save” the treatment until exhaustion of the prior claim’s days’ supply of 28 days). Since eptinezumab and onabotulinumtoxinA are administered in a healthcare provider’s office and a patient cannot “save” the treatment, early claims for these drugs were not adjusted.

### Statistical analysis

Baseline characteristics between the five treatment groups were compared using chi-square (or Fisher’s exact test for small sample sizes) for categorical variables and ANOVA for continuous variables (or Kruskal–Wallis for non–normally distributed continuous variables). The unadjusted probability of continuing the index therapy between treatment groups was compared using Kaplan–Meier curves with log-rank tests. A Cox proportional-hazards model with bootstrapped 95% confidence intervals was used to estimate the hazard of index drug discontinuation between treatment groups unadjusted and adjusted for covariates, with eptinezumab as the reference group. The proportionality of hazards assumption was verified using visual inspection of log(-log[survival]) versus log(time) plot and tested using Schoenfeld residuals. Due to violation of the proportionality assumption, 95% confidence intervals of model coefficients were estimated by bootstrapping methods.

### Sensitivity analysis

Two analyses were performed: a “most recent treatment episode” analysis to maximize eptinezumab sample size and a “new user” sensitivity analysis to support the robustness of the “most recent treatment episode” analysis. In the base case analysis, a treatment episode was defined as a period of treatment with the same drug without a ≥ 15-day gap in therapy. This was tested with a 30-day, 60-day, and 90-day treatment gap and independent of previous anti-CGRP use.

Since the results of the most recent treatment episode analysis were subject to a high risk of confounding due to the history of anti-CGRP mAb or onabotulinumtoxinA treatment and severity of disease, an analysis based on a “new user” cohort with no history of anti-CGRP mAb or onabotulinumtoxinA treatment was performed. The analysis followed the same methodology applied to the most recent treatment episode analysis. For this analysis, the overall study period began on June 25, 2019, and ended on December 31, 2021. The index period began June 25, 2020 (date of first eptinezumab claim) and ended on December 31, 2021. The period beginning June 25, 2019, served as the baseline period for measurement of baseline characteristics.

## Results

### Most recent treatment episode analysis

**Fig. 2:**
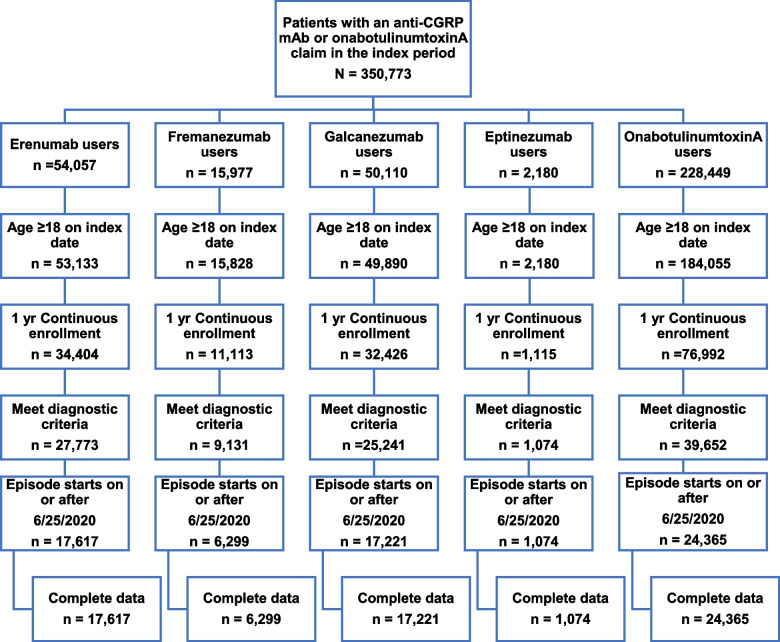
Patients identified to have a claim for an anti-CGRP mAb or onabotulinumtoxinA. Anti-CGRP mAb, anti-calcitonin gene-related peptide monoclonal antibody

During the index period (June 25, 2020–December 31, 2021), there were 350,773 patients with a claim for an anti-CGRP mAb or onabotulinumtoxinA. After application of inclusion criteria, the total sample size was 66,567 patients with migraine who had a most recent episode of treatment occurring after June 25, 2020 (Fig. 2). A greater proportion of patients on eptinezumab or onabotulinumtoxinA had a baseline diagnosis of CM as well as ≥ 3 comorbidities in the baseline period (Table 1). In evaluating medication histories, a greater proportion of patients on eptinezumab had used ≥ 3 lines of non–anti-CGRP oral preventives, ≥ 3 lines of acute therapies, and had prior exposure to oral anti-CGRP medications. Lastly, patients on eptinezumab had a higher number of treatment episodes prior to their most recent treatment episode.

**Table 1:** Baseline demographics

Variable	Level	Overall	Eptinezumab	Erenumab	Fremanezumab	Galcanezumab	OnabotulinumtoxinA
		***N*** = 66,576	***n*** = 1,074	***n*** = 17,617	***n*** = 6,299	***n*** = 17,221	***n*** = 24,365
**Age, median (Q1, Q3)**		46 (36, 54)	46 (38, 55)	45 (35, 53)	46 (36, 54)	45 (36, 53)	47 (37, 55)
**Sex, n (%)**	Female	58,983 (88.59)	929 (86.50)	15,402 (87.43)	5,545 (88.03)	15,101 (87.69)	22,006 (90.32)
	Male	7,593 (11.41)	145 (13.50)	2,215 (12.57)	754 (11.97)	2,120 (12.31)	2,359 (9.68)
**Payer type, n (%)**	Other	8,018 (12.04)	125 (11.64)	1,884 (10.69)	1,046 (16.61)	2,010 (11.67)	2,953 (12.12)
	HMO	10,041 (15.08)	180 (16.76)	2,457 (13.95)	1,026 (16.29)	2,534 (14.71)	3,844 (15.78)
	PPO	48,517 (72.87)	769 (71.60)	13,276 (75.36)	4,227 (67.11)	12,677 (73.61)	17,568 (72.10)
**Migraine type, n (%)**	Episodic	31,480 (47.28)	347 (32.31)	11,838 (67.20)	3,958 (62.84)	11,087 (64.38)	4,250 (17.44)
	Chronic	35,096 (52.72)	727 (67.69)	5,779 (32.80)	2,341 (37.16)	6,134 (35.62)	20,115 (82.56)
**Presence of select comorbidities, n (%)** ^a^	0	13,961 (20.97)	188 (17.50)	3,860 (21.91)	1,380 (21.91)	3,563 (20.69)	4,970 (20.40)
	1	14,946 (22.45)	210 (19.55)	4,017 (22.80)	1,498 (23.78)	3,890 (22.59)	5,331 (21.88)
	2	13,080 (19.65)	210 (19.55)	3,470 (19.70)	1,228 (19.50)	3,401 (19.75)	4,771 (19.58)
	≥ 3	24,589 (36.93)	466 (43.39)	6,270 (35.59)	2,193 (34.82)	6,367 (36.97)	9,293 (38.14)
**Prescriber specialty, n (%)**	Other	14,661 (22.02)	376 (35.01)	2,842 (16.13)	1,020 (16.19)	2,764 (16.05)	7,659 (31.43)
	Home Health	2,299 (3.45)	85 (7.91)	262 (1.49)	130 (2.06)	295 (1.71)	1,527 (6.27)
	ER	132 (0.20)	0 (0.00)	47 (0.27)	18 (0.29)	51 (0.30)	16 (0.07)
	GP/FP/Internist	9,851 (14.80)	47 (4.38)	3,728 (21.16)	1,018 (16.16)	3,640 (21.14)	1,418 (5.82)
	Hospitalist	3,074 (4.62)	192 (17.88)	851 (4.83)	273 (4.33)	774 (4.49)	984 (4.04)
	Neurologist	28,954 (43.49)	250 (23.28)	7,519 (42.68)	3021 (47.96)	7,211 (41.87)	10,953 (44.95)
	NP/PA	7,541 (11.33)	124 (11.55)	2,357 (13.38)	810 (12.86)	2,455 (14.26)	1,795 (7.37)
	Urgent Care	64 (0.10)	0 (0.00)	11 (0.06)	9 (0.14)	31 (0.18)	13 (0.05)
**History of oral non–anti-CGRP preventive therapy lines used, n (%)** ^b^	0	22,390 (33.63)	252 (23.46)	5,944 (33.74)	2,085 (33.10)	5,634 (32.72)	8,475 (34.78)
	1	19,486 (29.27)	256 (23.84)	5,347 (30.35)	1,905 (30.24)	5,271 (30.61)	6,707 (27.53)
	2	13,033 (19.58)	233 (21.69)	3,421 (19.42)	1,254 (19.91)	3,435 (19.95)	4,690 (19.25)
	≥ 3	11,667 (17.52)	333 (31.01)	2,905 (16.49)	1,055 (16.75)	2,881 (16.73)	4,493 (18.44)
**History of acute therapy lines used, n (%)** ^c^	0	23,384 (35.12)	286 (26.63)	6,047 (34.32)	2,129 (33.80)	5,762 (33.46)	9,160 (37.59)
	1	20,653 (31.02)	283 (26.35)	5,849 (33.20)	2,062 (32.74)	5,607 (32.56)	6,852 (28.12)
	2	11,348 (17.05)	182 (16.95)	3,002 (17.04)	1,080 (17.15)	3,108 (18.05)	3,976 (16.32)
	≥ 3	11,191 (16.81)	323 (30.07)	2,719 (15.43)	1,028 (16.32)	2,744 (15.93)	4,377 (17.96)
**Use of acute gepant, n (%)** ^d^	No	54,986 (82.59)	633 (58.94)	14,922 (84.70)	4,899 (77.77)	14,276 (82.90)	20,256 (83.14)
	Yes	11,590 (17.41)	441 (41.06)	2,695 (15.30)	1,400 (22.23)	2,945 (17.10)	4,109 (16.86)
**History of emergency department visits, median (Q1, Q3)**		0 (0, 0)	0 (0, 1)	0 (0, 0)	0 (0, 0)	0 (0, 0)	0 (0, 0)
**History of urgent care visits, median (Q1, Q3)**		0 (0, 0)	0 (0, 0)	0 (0, 0)	0 (0, 0)	0 (0, 0)	0 (0, 0)
**History of inpatient hospitalizations, median (Q1, Q3)**		0 (0, 0)	0 (0, 0)	0 (0, 0)	0 (0, 0)	0 (0, 0)	0 (0, 0)
**Charlson comorbidity index, median (Q1, Q3)**		0 (0, 0)	0 (0, 0)	0 (0, 0)	0 (0, 0)	0 (0, 0)	0 (0, 0)
**Copay ($ total patient paid), median (Q1, Q3)**		40 (0, 122)	0 (0, 312)	40 (9, 80)	45 (0, 122)	40 (0, 85)	0 (0, 225)
**# of episodes before index episode, median (Q1, Q3)**		1 (0, 3)	3 (2, 5)	1 (0, 3)	1 (0, 3)	1 (0, 2)	2 (1, 4)

^a^Comorbidities included: Depression, anxiety, sleep disorders, fibromyalgia, malaise/fatigue, hypertension, ischemic heart disease, cerebrovascular disease, overweight/obesity, and constipation

^b^Anticonvulsants, antidepressants (SNRIs, TCAs, SSRIs, MAOIs), antihistamines, antihypertensives (ACE inhibitors, ARBs, alpha agonists, beta blockers, calcium channel blockers), NMDA antagonists

^c^Acute therapy lines use: Triptans, analgesics (narcotic, non-narcotic, anti-inflammatory), hypnotics, ergots, ditans, isometheptene, and antiemetics

^d^Acute gepant lines include: Rimegepant (may be used acutely or for prevention) and ubrogepant

*ACE* Angiotensin-converting enzyme, *Anti-CGRP* Anti-calcitonin gene-related peptide, *ARBs* Angiotensin II receptor blockers, *ER* Emergency room, *FP* Family practitioner, *GP* General practitioner, *HMO* Health maintenance organization, *MAOIs* Monoamine oxidase inhibitors, *NMDA* N-methyl D-aspartate, *NP*, Nurse practitioner, *PA* Physician assistant, *PPO* Preferred provider organization, *SNRI* Serotonin and norepinephrine reuptake inhibitors, *SSRI* Selective serotonin reuptake inhibitors, *TCA* Tricyclic antidepressants

**Fig. 3:**
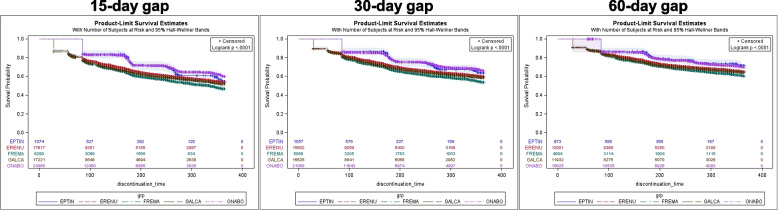
Most recent treatment episode: Unadjusted probability of remaining on treatment. Wilcoxon *p*-value < 0.0001. Note: sample size is reduced since extending the gap period changes the definition of an episode (e.g., distinct episodes with the same drug based on a 15-day gap may become a single episode and is excluded if the episode begins before June 25, 2020)

The probability of remaining on treatment across time was similar between eptinezumab and onabotulinumtoxinA, and persistence for both was significantly higher compared to erenumab, fremanezumab, and galcanezumab based on a 15-day treatment gap in the most recent treatment episode base case analysis. The difference in the probability of remaining on treatment was attenuated, but still significant, and overall persistence increased when extending the gap period to 30 or 60 days (Fig. 3). Results of a 90-day treatment gap post hoc analysis were consistent with the 30- and 60-day treatment gaps, where eptinezumab and onabotulinumtoxinA have similar discontinuation rates but are lower compared to SC anti-CGRP mAbs (Supplemental Fig. 1 and Supplemental Table 1). Unadjusted and adjusted Cox proportional hazards models yielded similar results; compared to eptinezumab, onabotulinumtoxinA had a similar rate of discontinuation, but SC anti-CGRP mAbs had a significantly higher rate of discontinuation (Table 2). Adjusted Cox proportional hazards models based on 30- and 60-day gaps also yielded similar results to the primary analysis based on a 15-day gap. The increased hazard of discontinuation among the SC anti-CGRP mAbs attenuated toward the null as the gap period was extended.

**Table 2:** Most recent treatment episode: Hazard ratio of treatment discontinuation

	**Cox Model Hazard Ratio** ^a^ **(95% confidence interval)** ^b^		
	**15-day gap** ***n*** = 66,576	**30-day gap** ***n*** = 59,441	**60-day gap** ***n*** = 45,125
**Erenumab**	1.32 (1.19, 1.49)	1.26 (1.11, 1.44)	1.19 (1.02, 1.40)
**Fremanezumab**	1.58 (1.42, 1.80)	1.53 (1.34, 1.75)	1.42 (1.22, 1.66)
**Galcanezumab**	1.42 (1.27, 1.61)	1.35 (1.19, 1.53)	1.26 (1.08, 1.47)
**OnabotulinumtoxinA**	0.98 (0.87, 1.10)	0.98 (0.87, 1.12)	1.02 (0.87, 1.18)
**Eptinezumab**	Reference	Reference	Reference

^a^Adjusted for all covariates (including age, sex, insurance type, migraine type, comorbidities, prescriber, prior anti-CGRP mAb and onabotulinumtoxinA treatment history, history of non–anti-CGRP oral preventives, history of acute medication use, and history of inpatient/outpatient/ER visits)

^b^Boot-strapped confidence interval. Note: sample size is reduced since extending the gap period changes the definition of an episode (e.g., distinct episodes with the same drug based on a 15-day gap may become a single episode and is excluded if the episode begins before June 25, 2020)

### New user sensitivity analysis

During the new user index period (June 25, 2020–December 31, 2021), after application of inclusion criteria and restriction to new users after June 25, 2020, a total of 30,507 patients were included in the cohort. A greater proportion of new users of eptinezumab and onabotulinumtoxinA had CM compared to the SC anti-CGRP mAbs. Moreover, compared to SC anti-CGRP mAbs, a greater proportion of patients on eptinezumab had ≥ 3 comorbidities, ≥ 3 prior lines of oral non–anti-CGRP preventive therapies, ≥ 3 prior lines of acute migraine medications, and had prior exposure to gepants (Supplemental Table 2).

**Fig. 4:**
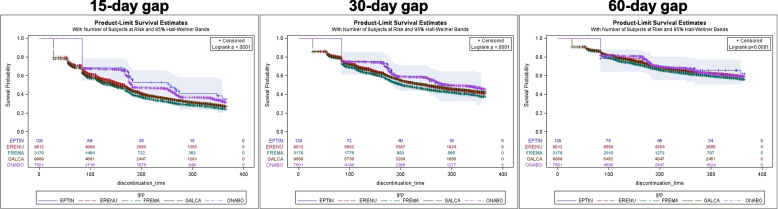
New users: Unadjusted probability of remaining on treatment. Wilcoxon *p*-value < 0.0001

The probability of remaining on treatment across time was similar between eptinezumab and onabotulinumtoxinA, but both were significantly higher compared to erenumab, fremanezumab, and galcanezumab. Upon extending the treatment gap to 30 and 60 days, the difference in the probability of remaining on treatment across time between groups diminished and became insignificant (Fig. 4). Compared to eptinezumab, onabotulinumtoxinA had a similar rate of discontinuation, but SC anti-CGRP mAbs had a significantly higher rate of discontinuation (Table 3). Based on a 15-day treatment gap, < 5% of patients discontinued and switched from their index therapy. Notably, only two patients were observed to discontinue and switch from eptinezumab (one to galcanezumab, the other to onabotulinumtoxinA). Among the other therapies, most patients who discontinued and switched therapies initiated a different SC anti-CGRP mAb or onabotulinumtoxinA. Upon extending the gap period to 30 and 60 days, the proportion of patients who switched decreased, with the majority of switchers initiating an anti-CGRP SC mAb or onabotulinumtoxinA (Supplemental Table 3).

**Table 3:** New users: Hazard ratio of treatment discontinuation

	**Cox Model Hazard Ratio** ^a^ **(95% confidence interval)** ^b^		
	**15-day gap** ***n*** = 30,507	**30-day gap** ***n*** = 30,507	**60-day gap** ***n*** = 30,507
**Erenumab**	1.48 (1.20, 1.90)	1.25 (0.99, 1.66)	1.08 (0.80, 1.54)
**Fremanezumab**	1.68 (1.36, 2.16)	1.45 (1.13, 1.95)	1.26 (0.92, 1.80)
**Galcanezumab**	1.55 (1.25, 1.97)	1.27 (0.99, 1.68)	1.11 (0.82, 1.59)
**OnabotulinumtoxinA**	1.17 (0.95, 1.49)	1.07 (0.84, 1.43)	1.09 (0.82, 1.54)
**Eptinezumab**	Reference	Reference	Reference

^a^Adjusted for all covariates (including age, sex, insurance type, migraine type, comorbidities, prescriber, history of non–anti-CGRP oral preventives, history of acute medication use, and history of inpatient/outpatient/ER visits)

^b^Boot-strapped confidence interval

## Discussion

The aim of this study was to compare the persistence of SC-delivered erenumab, fremanezumab, and galcanezumab; IV-delivered eptinezumab; or IM-delivered onabotulinumtoxinA in patients with migraine via a retrospective cohort study. Among patients with migraine, the rate of discontinuation of SC anti-CGRP mAbs was significantly higher compared to eptinezumab and onabotulinumtoxinA based on a 15-day treatment gap. This observation in the most recent treatment episode analysis was not sensitive to the length of the treatment gap, but the difference in the rate of discontinuation was attenuated when extending the treatment gap period to 30 days and to 60 days.

Although these data did not allow us to determine why patients switched/discontinued medications, the attenuation of the relative difference in the rate of discontinuation may be due to patients on SC anti-CGRP mAbs delaying their next prescription fill, potentially due to the availability of drug samples. Since eptinezumab and onabotulinumtoxinA are administered by clinicians, drug sampling rarely occurs. The longer gap (e.g., 90-day gap) analyses can account for any potential gaps in therapy due to medication sampling. This may have affected the persistence observed in shorter gap analyses (e.g., 15- or 30-day gaps) if a patient was filling an SC medication every 3 months rather than monthly because of medication sampling. Conversely, the persistence of SC treatment might be overestimated; patients who initiated an SC treatment with a medication sample and did not experience a response to therapy will not appear in this analysis as having discontinued treatment.

In a cohort of patients who newly initiated an anti-CGRP mAb or onabotulinumtoxinA, the rate of discontinuation of SC anti-CGRP mAbs was significantly higher compared to eptinezumab and onabotulinumtoxinA based on a 15-day treatment gap, but not according to a 30-day or 60-day gap. This contrasts with the results of the most recent treatment episode analysis in which the higher rate of discontinuation in patients on an anti-CGRP SC mAb remained significantly higher even when the treatment gap was extended.

Differing routes of administration and dosing frequency, such as monthly SC injections of erenumab, fremanezumab, and galcanezumab; quarterly SC injections of fremanezumab; quarterly IV infusions of eptinezumab; or quarterly IM injections of onabotulinumtoxinA to the face, head, and neck, may affect persistence to therapy. The lower rate of eptinezumab discontinuation among patients who have had prior treatment episodes with an anti-CGRP SC mAb or onabotulinumtoxinA may be a marker of treatment effectiveness or tolerability (i.e., a sign that eptinezumab may be effective where other therapies are not). Alternatively, this could simply reflect patient preferences for how treatment is administered or that they have tried all other treatment options available. Regardless of the gap period and type of cohort used, onabotulinumtoxinA had a similar rate of discontinuation compared to eptinezumab and was also lower compared to the anti-CGRP SC mAbs. This may indicate that the quarterly dosing interval and greater healthcare provider involvement in the administration of eptinezumab or onabotulinumtoxinA may contribute to continued persistency on therapy, in contrast to the self-administration of SC anti-CGRP mAbs.

Among new users, fewer than 5% of patients who discontinued a newly initiated anti-CGRP mAb or onabotulinumtoxinA subsequently initiated a new therapy. This may indicate that some patients with migraine may be unwilling to try other anti-CGRP mAbs or onabotulinumtoxinA after an initial treatment failure. When extending the gap period from 15 to 30 and 60 days, the proportion of patients who discontinued and switched decreased. As discussed above, this observation suggests that patients tend to delay their next medication claim or, for SC treatments, potentially had samples available.

Our results are supported by a similar retrospective cohort study evaluating persistence to onabotulinumtoxinA among patients with CM who newly initiated onabotulinumtoxinA or an anti-CGRP SC mAb in the IBM MarketScan Commercial and Medicare Supplemental database [12]. The study found that at 6 months after initiating onabotulinumtoxinA, persistence was 67% compared to 46% for SC anti-CGRP mAbs based on a 30-day treatment gap (*P* < 0.001). Our study also found that persistence to onabotulinumtoxinA was numerically higher compared to SC anti-CGRP mAbs. Among new users of onabotulinumtoxinA, the proportion of patients remaining on therapy at 6 months based on a 30-day treatment gap was about 60%, whereas the proportion of patients remaining on SC anti-CGRP mAb therapy was about 50%.

### Limitations

The results of this study must be interpreted considering the limitations of the methodology. Only patients with commercial insurance were included in this analysis, which excludes a portion of the migraine population with other insurance coverage types. It may be speculated that the median copay of $0 for eptinezumab and onabolulinumtoxinA contributed to the longer continuation of use compared to the other anti-CGRP mAbs. However, there are other factors to consider, such as differences in insurance type (which is adjusted for in the hazard model), and the fact that for patients who receive eptinezumab or onabotulinumtoxinA, they must travel and potentially pay additional fees at the facility where treatment is rendered. This may actually lead to sooner discontinuation. The diagnosis of migraine was based on inpatient and outpatient claims, which is subject to misclassification. Of note, the timing when each drug was released may have affected classification. Moreover, for onabotulinumtoxinA, the drug is only approved for CM; therefore, in theory, the cohort should be 100% CM. It may be possible that patients transitioned from CM to episodic migraine (EM) and kept using onabotulinumtoxinA treatment; alternatively, an original EM diagnosis may not have been updated despite the patient receiving onabotulinumtoxinA. In this data analysis, the reason for discontinuation or switching of therapy is unknown as claims data do not provide this information. More research and data are needed to better understand the rules of payors and their role in the discontinuation of anti-CGRP therapy for migraine care, as well as other factors for discontinuation. Patients may receive samples of drugs or face drug prior-authorization barriers, which may lead to gaps in therapy but not necessarily a discontinuation. Additionally, onabotulinumtoxinA and oral preventive medications are indicated for other conditions, and their use may not be for migraine despite that the patient has a migraine diagnosis. Moreover, there are many treatment steps required before a patient can access eptinezumab, which may reflect why fewer patients are in this group. Persistence was based on claims data and may not reflect actual medication-taking behavior. If patients dropped off insurance or switched insurance plans, we would not be able to observe all claims and therefore, patients were censored. Sometimes, patients are forced to switch between anti-CGRP SC mAbs for non-medical reasons (i.e., formulary or insurance plan changes). Lastly, unobserved confounders such as migraine frequency and severity may confound the association between treatment and persistence to treatment.

## Conclusions

Adherence to preventive medications is often predicated on efficacy, tolerability, and patient satisfaction. Based on a 15-day treatment gap, persistence to eptinezumab was similar to onabotulinumtoxinA, and persistence to either therapy was superior to SC anti-CGRP mAbs among patients with a history of anti-CGRP mAb or onabotulinumtoxinA use. While most patients receiving eptinezumab or onabotulinumtoxinA have CM, the statistical model includes diagnosis (i.e., EM vs CM) as a covariate. This suggests that eptinezumab and onabotulinumtoxinA may have superior efficacy and tolerability in the population of patients with CM.

## Supplementary Information

**Additional file 1: Supplemental Figure 1. **Most recent treatment episode: Unadjusted probability of remaining on treatment (90-day gap). **Supplemental Table 1.** Most recent treatment episode: Hazard ratio of treatment discontinuation (90-day gap). **Supplemental Table 2.** Baseline characteristics (new users). **Supplemental Table 3. **Drug switching in new users.

## Data Availability

The data sets generated and/or analyzed during the current study are not publicly available due to contractual restrictions by the data owner (IQVIA).

## Abbreviations

CM: Chronic migraine
CGRP: Calcitonin gene-related peptide
EM: Episodic migraine
ICD: International Classification of Diseases
IM: Intramuscular
IV: Intravenous
mAbs: Monoclonal antibodies
SC: Subcutaneous

## References

[1] Ailani J, Burch RC, Robbins MS, on behalf of the Board of Directors of the American Headache Society (2021). The American Headache Society Consensus Statement: Update on integrating new migraine treatments into clinical practice. Headache.

[2] Woolley JM, Bonafede MM, Maiese BA, Lenz RA (2017). Migraine prophylaxis and acute treatment patterns among commercially insured patients in the United States. Headache.

[3] Cramer JA, Roy A, Burrell A, Fairchild CJ, Fuldeore MJ, Ollendorf DA, Wong PK (2008). Medication compliance and persistence: terminology and definitions. Value Health.

[4] Blumenfeld AM, Bloudek LM, Becker WJ, Buse DC, Varon SF, Maglinte GA, Wilcox TK, Kawata AK, Lipton RB (2013). Patterns of use and reasons for discontinuation of prophylactic medications for episodic migraine and chronic migraine: results from the second international burden of migraine study (IBMS-II). Headache.

[5] Botox [package insert]. In:Allergan, Madison, NJ. (2021)

[6] VYEPTI [package insert]. In:Lundbeck Seattle BioPharmaceuticals, Inc., Bothell, WA. (2021)

[7] AJOVY [package insert]. In:Teva Pharmaceuticals USA, Inc., North Wales, PA. (2020)

[8] EMGALITY [package insert]. In:Eli Lilly and Company, Indianapolis, IN. (2019)

[9] AIMOVIG [package insert]. In:Amgen Inc., Thousand Oaks, CA. (2020)

[10] Diener HC, Dodick DW, Aurora SK, Turkel CC, DeGryse RE, Lipton RB, Silberstein SD, Brin MF (2010). OnabotulinumtoxinA for treatment of chronic migraine: results from the double-blind, randomized, placebo-controlled phase of the PREEMPT 2 trial. Cephalalgia.

[11] Tepper SJ, Fang J, Vo P, Shen Y, Zhou L, Abdrabboh A, Glassberg M, Ferraris M (2021). Impact of erenumab on acute medication usage and health care resource utilization among migraine patients: a US claims database study. J Headache Pain.

[12] Schwedt TJ LJ, Knievel K (2022). Real-World Persistence and Costs Among Patients With Chronic Migraine Treated With OnabotulinumtoxinA or CGRP mAbs: A Retrospective Claims Analysis Study (P10–2.004). Neurology.

