# Supplementary material for: Persistence to anti-CGRP monoclonal antibodies and onabotulinumtoxinA among patients with migraine: a retrospective cohort study
Source: J Headache Pain. 2023 Aug 2;24(1):101. doi: 10.1186/s10194-023-01636-8 (PMC10394944; doi:10.1186/s10194-023-01636-8)
Supplement: Supplementary file 1 — Additional file 1: Supplemental Figure 1. Most recent treatment episode: Unadjusted probability of remaining on treatment (90-day gap). Supplemental Table 1. Most recent treatment episode: Hazard ratio of treatment discontinuation (90-day gap). Supplemental Table 2. Baseline characteristics (new users). Supplemental Table 3. Drug switching in new users. [file 10194_2023_1636_MOESM1_ESM.pdf]

Supplemental Material

Supplemental Fig 1. Most recent treatment episode: Unadjusted probability of remaining on treatment (90-day gap)

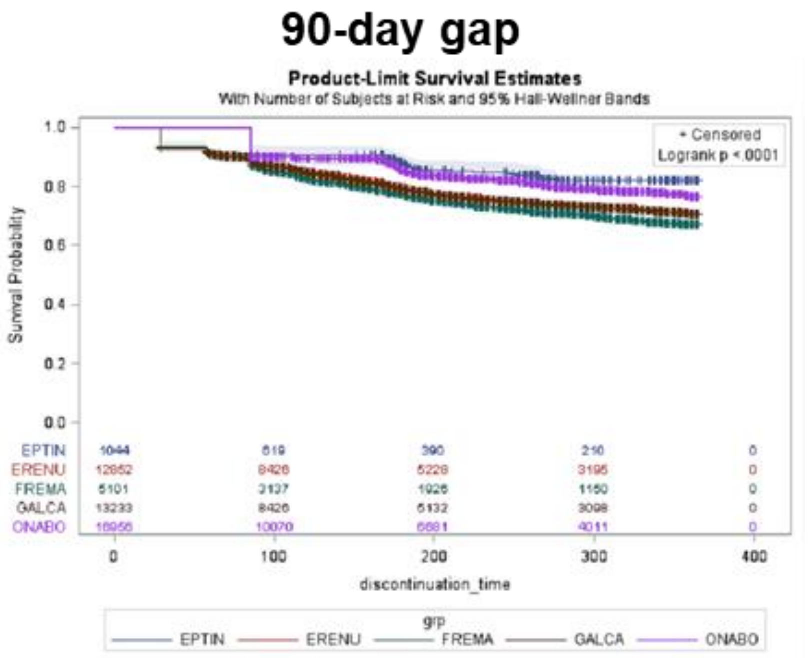

Supplemental Table 1. Most recent treatment episode: Hazard ratio of treatment discontinuation (90-day gap)

	**CM Hazard Ratio*** **(95% confidence interval)**†
**Erenumab**	1.412 (1.184, 1.684)
**Fremanezumab**	1.673 (1.396, 2.004)
**Galcanezumab**	1.432 (1.201, 1.707)
**OnabotulinumtoxinA**	1.120 (0.941, 1.333)
**Eptinezumab**	Reference

*Adjusted for age, sex, payer type, comorbidities, prescriber, index drug co-pay, number of prior anti-CGRP or botulinumtoxin episdoes, history of non–anti-CGRP oral preventive medication use, history of acute medication use, gepant use, history of ED/urgent care/hospitalizations. †Boot-strapped confidence interval.

Supplemental Table 2. Baseline characteristics (new users)

Variable	Level	Overall	Eptinezumab	Erenumab	Fremanezumab	Galcanezumab	OnabotulinumtoxinA
		N=30,507	n=126	n=9,812	n=3,179	n=9,889	n=7,501
**Age, median (Q1, Q3)**		43 (34, 52)	46 (36, 56)	43 (33, 52)	43 (34, 52)	43 (34, 52)	44 (35, 53)
**Sex, n (%)**	Female	26,797 (87.84)	109 (86.51)	8,586 (87.51)	2,769 (87.10)	8,653 (87.50)	6,680 (89.05)
	Male	3,710 (12.16)	17 (13.49)	1,226 (12.49)	410 (12.90)	1,236 (12.50)	821 (10.95)
**Payer type, n (%)**	Other	3,544 (11.62)	15 (11.90)	970 (9.89)	505 (15.89)	1,107 (11.19)	947 (12.62)
	HMO	4,498 (14.74)	18 (14.29)	1,350 (13.76)	551 (17.33)	1,397 (14.13)	1,182 (15.76)
	PPO	22,465 (73.64)	93 (73.81)	7,492 (76.36)	2,123 (66.78)	7,385 (74.68)	5,372 (71.62)
**Migraine type, n (%)**	Episodic	17,226 (56.47)	37 (29.37)	6,840 (69.71)	2,131 (67.03)	6,569 (66.43)	1,649 (21.98)
	Chronic	13,281 (43.53)	89 (70.63)	2,972 (30.29)	1,048 (32.97)	3,320 (33.57)	5,852 (78.02)
**Presence of select comorbidities, n (%)***	0	6,680 (21.90)	19 (15.08)	2,213 (22.55)	761 (23.94)	2,189 (22.14)	1,498 (19.97)
	1	7,046 (23.10)	26 (20.63)	2,265 (23.08)	773 (24.32)	2,303 (23.29)	1,679 (22.38)
	2	6,034 (19.78)	29 (23.02)	1,954 (19.91)	627 (19.72)	1,922 (19.44)	1,502 (20.02)
	≥3	10,747 (35.23)	52 (41.27)	3,380 (34.45)	1,018 (32.02)	3,475 (35.14)	2,822 (37.62)
**Prescriber specialty, n (%)**	Other	5,540 (18.16)	50 (39.68)	1,436 (14.64)	455 (14.31)	1,464 (14.80)	2,135 (28.46)
	Home Health	777 (2.55)	13 (10.32)	148 (1.51)	65 (2.04)	146 (1.48)	405 (5.40)
	ER	66 (0.22)	0 (0.00)	22 (0.22)	8 (0.25)	29 (0.29)	7 (0.09)
	GP/FP/Internist	5,207 (17.07)	4 (3.17)	2,038 (20.77)	528 (16.61)	2,083 (21.06)	554 (7.39)
	Hospitalist	1,288 (4.22)	22 (17.46)	395 (4.03)	93 (2.93)	392 (3.96)	386 (5.15)
	Neurologist	13,982 (45.83)	25 (19.84)	4,524 (46.11)	1,637 (51.49)	4,377 (44.26)	3,419 (45.58)
	NP/PA	3,608 (11.83)	12 (9.52)	1,241 (12.65)	387 (12.17)	1,378 (13.93)	590 (7.87)
	Urgent Care	39 (0.13)	0 (0.00)	8 (0.08)	6 (0.19)	20 (0.20)	5 (0.07)
**History of oral non–anti-CGRP preventive therapy lines used, n (%)†**	0	10,243 (33.58)	43 (34.13)	3,213 (32.75)	1,075 (33.82)	3,197 (32.33)	2,715 (36.20)
	1	9,080 (29.76)	31 (24.60)	2,963 (30.20)	968 (30.45)	3,058 (30.92)	2,060 (27.46)
	2	6,263 (20.53)	20 (15.87)	2,041 (20.80)	662 (20.82)	2,057 (20.80)	1,483 (19.77)
	≥3	4,921 (16.13)	32 (25.40)	1,595 (16.26)	474 (14.91)	1,577 (15.95)	1,243 (16.57)
**History of acute therapy lines used, n (%)‡**	0	109,21 (35.80)	53 (42.06)	3,354 (34.18)	1,138 (35.80)	3,326 (33.63)	3,050 (40.66)
	1	9,723 (31.87)	24 (19.05)	3,320 (33.84)	1,057 (33.25)	3,236 (32.72)	2,086 (27.81)
	2	5,266 (17.26)	20 (15.87)	1,704 (17.37)	528 (16.61)	1,809 (18.29)	1,205 (16.06)
	≥3	4,597 (15.07)	29 (23.02)	1,434 (14.61)	456 (14.34)	1,518 (15.35)	1,160 (15.46)
**Use of acute gepants, n (%)§**	No	27,074 (88.75)	98 (77.78)	8,833 (90.02)	2,693 (84.71)	8,712 (88.10)	6,738 (89.83)
	Yes	3,433 (11.25)	28 (22.22)	979 (9.98)	486 (15.29)	1,177 (11.90)	763 (10.17)
**History of emergency department visits, median (Q1, Q3)**		0 (0, 0)	0 (0, 1)	0 (0, 0)	0 (0, 0)	0 (0, 0)	0 (0, 1)

**History of urgent care visits, median (Q1, Q3)**		0 (0, 0)	0 (0, 0)	0 (0, 0)	0 (0, 0)	0 (0, 0)	0 (0, 0)
**History of inpatient hospitalizations, median (Q1, Q3)**		0 (0, 0)	0 (0, 0)	0 (0, 0)	0 (0, 0)	0 (0, 0)	0 (0, 0)
**Charlson comorbidity index, median (Q1, Q3)**		0 (0, 0)	0 (0, 0)	0 (0, 0)	0 (0, 0)	0 (0, 0)	0 (0, 0)
**Copay ($ total patient paid), median (Q1, Q3)**		45 (12, 159)	0 (0, 312)	45 (25, 120)	60 (30, 192)	45 (25, 125)	40 (0, 260)

[*]Comorbidities included: Depression, anxiety, sleep disorders, fibromyalgia, malaise/fatigue, hypertension, ischemic heart disease, cerebrovascular disease, overweight/obesity, and constipation. [†]Anticonvulsants, antidepressants (SNRIs, TCAs, SSRIs, MAOIs), antihistamines, antihypertensives (ACE inhibitors, ARBs, alpha agonists, beta blockers, calcium channel blockers), NMDA antagonists. [‡]Acute therapy lines use: Triptans, analgesics (narcotic, non-narcotic, anti-inflammatory), hypnotics, ergots, ditans, isometheptene, and antiemetics. [§]Acute gepant lines include: Rimegepant (may be used acutely or for prevention) and ubrogepant.

ACE; angiotensin-converting enzyme; Anti-CGRP, anti-calcitonin gene-related peptide; ARBs, angiotensin II receptor blockers; ER, emergency room; FP, family practitioner; GP, general practitioner; HMO, health maintenance organization; MAOIs, monoamine oxidase inhibitors; NMDA, N-methyl D-aspartate; NP, nurse practitioner; PA, physician assistant; PPO, preferred provider organization; SNRI, serotonin and norepinephrine reuptake inhibitors; SSRI, selective serotonin reuptake inhibitor; TCA, tricyclic antidepressants.

Supplemental Table 3. Drug switching in new users

15-day gap				Switched to drug*									
				Eptinezumab		Erenumab		Fremanezumab		Galcanezumab		OnabotulinumtoxinA	
Index drug	N	% Switched	# of Switchers	n	% of switchers	n	% of switchers	n	% of switchers	n	% of switchers	n	% of switchers
**Eptinezumab**	126	1.59	2	na		0	0.0	0	0.0	1	50.0	1	50.0
**Erenumab**	9,812	4.31	423	4	0.95	na		75	17.7	198	46.8	146	34.5
**Fremanezumab**	3,179	4.84	154	1	0.65	47	30.5	na		58	37.7	48	31.2
**Galcanezumab**	9,889	3.54	350	3	0.86	143	40.9	69	19.7	na		135	38.6
**OnabotulinumtoxinA**	7,501	3.44	258	6	2.33	96	37.2	40	15.5	116	45.0	na	

30-day gap				Switched to drug*									
				Eptinezumab		Erenumab		Fremanezumab		Galcanezumab		OnabotulinumtoxinA	
Index drug	N	% Switched	# of Switchers	n	% of switchers	n	% of switchers	n	% of switchers	n	% of switchers	n	% of switchers
**Eptinezumab**	126	1.59	2	na		0	0.0	0	0.0	1	50.0	1	50.0
**Erenumab**	9,812	3.96	389	3	0.8	na		65	16.7	204	52.4	117	30.1
**Fremanezumab**	3,179	4.28	136	0	0.0	43	31.6	na		56	41.2	37	27.2
**Galcanezumab**	9,889	3.28	324	2	0.6	147	45.4	74	22.8	na		101	31.2
**OnabotulinumtoxinA**	7,501	2.91	218	3	1.4	78	35.8	41	18.8	96	44.0	na	

60-day gap				Switched to drug*									
				Eptinezumab		Erenumab		Fremanezumab		Galcanezumab		OnabotulinumtoxinA	
Index drug	N	% Switched	# of Switchers	n	% of switchers	n	% of switchers	n	% of switchers	n	% of switchers	n	% of switchers
**Eptinezumab**	126	0.00	0	na		0	0.0	0	0.0	0	0.0	0	0.0
**Erenumab**	9,812	2.38	234	0	0.0	na		59	25.2	167	71.4	8	3.4
**Fremanezumab**	3,179	2.77	88	0	0.0	36	40.9	na		48	54.5	4	4.5
**Galcanezumab**	9,889	2.01	199	0	0.0	124	62.3	67	33.7	na		8	4.0
**OnabotulinumtoxinA**	7,501	1.64	123	1	0.8	41	33.3	19	15.4	62	50.4	na	

*Switch to this drug after discontinuation of index drug (–30 days to +90 days after discontinuation date, defined as fill date + days' supply); % of switchers is the percent of patients who switched to a particular drug out of the total number of patients who switched from the index drug. na, not applicable.
